# Precise large deviations for widely orthant dependent random variables with different distributions

**DOI:** 10.1186/s13660-018-1613-2

**Published:** 2018-01-16

**Authors:** Miaomiao Gao, Kaiyong Wang, Lamei Chen

**Affiliations:** School of Mathematics and Physics, Suzhou University of Science and Technology, Suzhou, 215009 P.R. China

**Keywords:** 60F10, 62E20, precise large deviations, widely orthant dependent, different distributions, dominantly varying tails

## Abstract

Let $X_{i}, i\geq1$ be a sequence of random variables with different distributions $F_{i}, i\geq1$. The partial sums are denoted by $S_{n}=\sum_{i=1}^{n} X_{i}$, $n\geq1$. This paper mainly investigates the precise large deviations of $S_{n}, n\geq1$, for the widely orthant dependent random variables $X_{i}, i\geq1$. Under some mild conditions, the lower and upper bounds of the precise large deviations of the partial sums $S_{n}$, $n\geq1$, are presented.

## Introduction

Let $X_{i}\ ( i\geq1 )$ and *X* be real-valued random variables (r.v.s) with distributions $F_{i}\ ( i\geq1 )$ and *F* and finite means $\mu_{i} \ ( i\geq1 )$ and *μ*, respectively. Let $S_{n}=\sum_{i=1}^{n}X_{i}$, $n\geq1$, be the partial sums. This paper investigates the precise large deviations for these partial sums $S_{n}$, $n\geq1$. That is to say, the paper studies the asymptotics of $P(S_{n}-E(S_{n})>x)$, which holds uniformly for all $x\geq\gamma n$ for every fixed $\gamma>0$ as *n* tends to ∞. In order to give the main results of this paper, we will introduce some notions and notation.

For a proper distribution *V* on $(-\infty,\infty)$, let ${\overline{V}}=1-V$ be its tail. Throughout this paper, all limit relations without explicit limit procedure are with respect to $n\rightarrow \infty$. For two positive functions $a(x)$ and $b(x)$, we write $a(x)=o(b(x))$ if $\lim_{x\to\infty} a(x)/b(x)=0$ and write $a(x)=O(b(x))$ if $\limsup_{x\to\infty} a(x)/b(x)<\infty$. ${\mathbf {1}}_{A}$ is the indicator function of the event *A*. For a real-valued number *c*, let $c^{+}=\max\{0, c\}$ and $c^{-}=-\min\{0,c\}$.

In this paper, we consider the random variables with heavy-tailed distributions. Some subclasses of heavy-tailed distribution classes will be introduced in the following. If for all $\beta>0$,

$$\int_{-\infty}^{\infty} e^{\beta x}V(d x)=\infty, $$

we say that the r.v. *ξ* (or its corresponding distribution *V*) is heavy-tailed; otherwise, the r.v. *ξ* (or *V*) is called light-tailed. A subclass of heavy-tailed distribution class is the class $\mathscr {D}$, which consists of all distributions with dominantly varying tails. Say that a distribution *V* on $(-\infty,\infty)$ belongs to the class $\mathscr{D}$ if, for any $y\in(0,1)$,

$$\limsup_{x\to\infty}\frac{{\overline{V}}(xy)}{{\overline{V}}(x)}< \infty. $$

Another slightly smaller class is the class $\mathscr{C}$, which consists of all distributions with consistently varying tails. We say that a distribution *V* on $(-\infty,\infty)$ belongs to the class $\mathscr{C}$ if

$$\lim_{y\nearrow1}\limsup_{x\to\infty}\frac{{\overline{V}}(xy)}{{\overline{V}}(x)}=1,\quad \mbox{or, equivalently,}\quad \lim_{y\searrow1}\liminf_{x\to\infty} \frac{{\overline{V}}(xy)}{{\overline{V}}(x)}=1. $$

A subclass of the class $\mathscr{C}$ is the class of distributions with regularly varying tails. A distribution *V* on $(-\infty,\infty )$ is said to be regularly varying at infinity with index *α*, denoted by $V\in\mathscr{R}_{-\alpha}$, if

$$\lim_{x\to\infty}\frac{\overline{V}(xy)}{\overline {V}(x)}=y^{-\alpha} $$

holds for some $0\leq\alpha<\infty$ and all $y>0$ (see, e.g., Bingham et al. [1]).

For a distribution *V*, denote the upper Matuszewska index of *V* by

$$\begin{aligned} &J^{+}_{V}=-\lim_{y\to\infty}\frac{\log{\overline{V}}_{*}(y)}{\log y}, \quad\mbox{with } {\overline{V}}_{*}(y):=\liminf_{x\to\infty}\frac{{\overline{V}}(xy)}{{\overline{V}}(x)}, y>1. \end{aligned}$$

Let $L_{V}=\lim_{y\searrow1}{\overline{V}}_{*}(y)$. From Chapter 2.1 of Bingham et al. [1], we know that the following assertions are equivalent:

$$(\mathrm{i}) \quad V\in\mathscr{D};\qquad (\mathrm{ii})\quad 0< L_{V}\leq1; \qquad( \mathrm{iii})\quad J^{+}_{V}< \infty. $$

From the definition of the class $\mathscr{C}$, it holds that $V\in \mathscr{C}$ if and only if $L_{V}=1$.

When $\{X_{i}, i\geq1\}$ are independent and identically distributed r.v.s, some studies of the precise large deviations of the partial sums $S_{n}, n\geq1$, can be found in Cline and Hsing [2], Heyde [3, 4], Heyde [5], Mikosch and Nagaev [6], Nagaev [7], Nagaev [8], Ng et al. [9] and so on. In Paulauskas and Skučaitė [10] and Skučaitė [11], the precise large deviations of a sum of independent but not identically distributed random variables were investigated. This paper considers the dependent r.v.s with different distributions. We investigate the r.v.s with the wide dependence structure, which is introduced in Wang et al. [12].

### Definition 1.1

For the r.v.s $\{\xi_{n}, n\geq1\}$, if there exists a finite real sequence $\{g_{U}(n), n\geq1\}$ satisfying, for each integer $n\geq1$ and for all $x_{i}\in(-\infty, \infty)$, $1\leq i\leq n$,

1.1 $$ P \Biggl(\bigcap^{n}_{i=1}\{ \xi_{i} > x_{i}\} \Biggr)\leq g_{U}(n)\prod _{i=1}^{n} P(\xi_{i}> x_{i}), $$

then we say that the r.v.s ${\{\xi_{n}, n\geq1\}}$ are widely upper orthant dependent (WUOD) with dominating coefficients $g_{U}(n), n\geq 1$; if there exists a finite real sequence $\{g_{L}(n), n\geq1\}$ satisfying, for each integer $n\geq1$ and for all $x_{i}\in(-\infty, \infty)$, $1\leq i\leq n$,

1.2 $$ P \Biggl(\bigcap^{n}_{i=1}\{ \xi_{i} \leq x_{i}\} \Biggr)\leq g_{L}(n)\prod _{i=1}^{n} P(\xi_{i}\leq x_{i}), $$

then we say that the r.v.s ${\{\xi_{n}, n\geq1\}}$ are widely lower orthant dependent (WLOD) with dominating coefficients $g_{L}(n), n\ge1$; if they are both WUOD and WLOD, then we say that the r.v.s ${\{\xi_{n}, n\geq1\}}$ are widely orthant dependent (WOD).

Definition 1.1 shows that the wide dependence structure contains the commonly used notions of the negatively upper/lower orthant dependence (see Ebrahimi and Ghosh [13] and Block et al. [14]) and the extendedly negatively orthant dependence (see Liu [15], Chen et al. [16] and Shen [17]). Here, we present an example of WOD r.v.s, which is the example of Wang et al. [12].

### Example 1.1

Assume that the random vectors $(\xi _{n},\eta_{n}), n=1,2,\ldots$ , are independent and, for each integer $n\geq1$, the r.v.s $\xi_{n}$ and $\eta_{n}$ are dependent according to the Farlie-Gumbel-Morgenstern copula with the parameter $\theta_{n}\in[-1,1]$:

$$C_{\theta_{n}}(u,v)=uv+\theta_{n}uv(1-u) (1-v), \quad (u,v) \in[0,1]^{2}, $$

which is absolutely continuous with density

$$c_{\theta_{n}}(u,v)=\frac{\partial^{2}C_{\theta_{n}}(u,v)}{ \partial u\,\partial v}=1+\theta_{n}(1-2u) (1-2v),\quad (u,v)\in[0,1]^{2} $$

(see, e.g., Example 3.12 in Nelsen [18]).

Suppose that the distributions of $\xi _{n}$ and $\eta_{n}, n=1, 2,\ldots$ , are absolutely continuous, denoted by $F_{\xi_{n}}$ and $F_{\eta_{n}}, n=1,2,\ldots$ , respectively. Hence, by Sklar’s theorem (see, e.g., Chapter 2 of Nelsen [18]), for each integer $n\geq1$ and any $x_{n},~y_{n}\in(-\infty,\infty)$,

$$\begin{aligned} P(\xi_{n}\leq x_{n}, \eta_{n}\leq y_{n})&= C_{\theta_{n}} \bigl(F_{\xi _{n}}(x_{n}),F_{\eta_{n}}(y_{n}) \bigr) \\ &=F_{\xi_{n}}(x_{n})F_{\eta_{n}}(y_{n}) \bigl(1+ \theta_{n}\overline{F_{\xi _{n}}}(x_{n}) \overline{F_{\eta_{n}}}(y_{n}) \bigr) \end{aligned}$$

and

$$\begin{aligned} P(\xi_{n}> x_{n}, \eta_{n}>y_{n}) &= \int^{1}_{F_{\xi_{n}}(x_{n})} \int ^{1}_{F_{\eta_{n}}(y_{n})} c_{\theta_{n}}(u,v)\,du\,dv \\ &=\overline{F_{\xi_{n}}}(x_{n})\overline{F_{\eta_{n}}}(y_{n}) \bigl(1+\theta_{n}F_{\xi_{n}}(x_{n})F_{\eta_{n}}(y_{n}) \bigr). \end{aligned}$$

Therefore, for each $n\geq1$, we have

$$\begin{aligned} a(\theta_{n})&:= \sup_{x_{n},y_{n}\in(-\infty,\infty)}\frac{P(\xi _{n}\leq x_{n}, \eta_{n}\leq y_{n})}{P(\xi_{n}\leq x_{n}) P(\eta_{n}\leq y_{n})} \\ &= \sup_{x_{n},y_{n}\in(-\infty,\infty)}\frac{P(\xi_{n}> x_{n}, \eta_{n}> y_{n})}{P(\xi_{n}> x_{n}) P(\eta_{n}> y_{n})} \\ &= \textstyle\begin{cases}1+\theta_{n} & 0< \theta_{n}\leq1; \\ 1 & -1\leq\theta_{n}\leq0, \end{cases}\displaystyle \end{aligned}$$

where, by convention, $\frac{0}{0}=1$. Thus, for each integer $n\geq 1$, we have

$$\begin{aligned} & \sup_{x_{i},y_{i}\in(-\infty,\infty),i=1,\ldots,n}\frac{P(\xi _{1}\leq x_{1},\eta_{1}\leq y_{1},\ldots,\xi_{n}\leq x_{n},\eta_{n}\leq y_{n})}{ \prod_{i=1}^{n}P(\xi_{i}\leq x_{i})P(\eta_{i}\leq y_{i})} \\ &\quad= \sup_{x_{i},y_{i}\in(-\infty,\infty),i=1,\ldots,n}\frac{\prod_{i=1}^{n}P(\xi_{i}\leq x_{i},\eta_{i}\leq y_{i})}{ \prod_{i=1}^{n}P(\xi_{i}\leq x_{i})P(\eta_{i}\leq y_{i})}=\prod _{i=1}^{n}a(\theta_{i}) \end{aligned}$$

and

$$\begin{aligned} & \sup_{x_{i},y_{i}\in(-\infty,\infty),i=1,\ldots,n}\frac{P(\xi _{1}\leq x_{1},\eta_{1}\leq y_{1},\ldots,\xi_{n}\leq x_{n})}{ \prod_{i=1}^{n-1}P(\xi_{i}\leq x_{i})P(\eta_{i}\leq y_{i})P(\xi_{n}\leq x_{n})} \\ &\quad= \sup_{x_{i},y_{i}\in(-\infty,\infty),i=1,\ldots,n}\frac{\prod_{i=1}^{n-1}P(\xi_{i}\leq x_{i},\eta_{i}\leq y_{i})}{ \prod_{i=1}^{n-1}P(\xi_{i}\leq x_{i})P(\eta_{i}\leq y_{i})}=\prod _{i=1}^{n-1}a(\theta_{i}), \end{aligned}$$

where, by convention, $\prod_{i=1}^{0}=1$. Similarly, for each integer $n\geq1$, we have

$$\begin{aligned} \sup_{x_{i},y_{i}\in(-\infty,\infty),i=1,\ldots,n}\frac{P(\xi_{1}> x_{1},\eta_{1}> y_{1},\ldots,\xi_{n}> x_{n},\eta_{n}> y_{n})}{ \prod_{i=1}^{n}P(\xi_{i}> x_{i})P(\eta_{i}> y_{i})}=\prod _{i=1}^{n}a(\theta _{i}) \end{aligned}$$

and

$$\begin{aligned} \sup_{x_{i},y_{i}\in(-\infty,\infty),i=1,\ldots,n}\frac{P(\xi_{1}> x_{1},\eta_{1}> y_{1},\ldots,\xi_{n}> x_{n})}{ \prod_{i=1}^{n-1}P(\xi_{i}> x_{i})P(\eta_{i}> y_{i})P(\xi_{n}> x_{n})} =\prod _{i=1}^{n-1}a(\theta_{i}). \end{aligned}$$

Hence, for the r.v.s $\xi_{1}, \eta_{1},\ldots, \xi_{n}, \eta_{n}, \ldots $ , we can take

$$g_{L}(n)=g_{U}(n)= \textstyle\begin{cases} \prod_{i=1}^{m}a(\theta_{i}) & n=2m, \\ \prod_{i=1}^{m-1}a(\theta_{i}) & n=2m-1, \end{cases}\displaystyle \quad m\geq1, $$

which makes relations (1.1) and (1.2) be satisfied. That is to say, the r.v.s $\xi_{1}, \eta_{1},\ldots$ , $\xi_{n}, \eta_{n}, \ldots$ , are WLOD and WUOD.

The wide dependent structure has been applied to many fields such as risk theory (see, e.g., Liu et al. [19], Wang et al. [20], Wang et al. [12], Mao et al. [21]), renewal theory (see, e.g., Wang and Cheng [22], Chen et al. [23]), complete convergence (Wang and Cheng [22], Qiu and Chen [24], Wang et al. [25], Chen et al. [23]), precise large deviations (see, e.g., Wang et al. [26], He et al. [27]) and some statistic fields (see, e.g., Wang and Hu [28]).

Wang et al. [12] gave the following properties of the wide dependent r.v.s.

### Proposition 1.1

(1) *Let* ${\{\xi_{n}, n\geq1\}}$ *be WLOD* (*WUOD*) *with dominating coefficients* $g_{L}(n)(g_{U}(n)), n\geq1$. *If* $\{f_{n}(\cdot), n\geq1\}$ *are nondecreasing*, *then* $\{f_{n}(\xi_{n}), n\geq1\}$ *are still WLOD* (*WUOD*) *with dominating coefficients* $g_{L}(n)(g_{U}(n)), n\geq1$. *If* $\{ f_{n}(\cdot), n\geq1\}$ *are nonincreasing*, *then* $\{f_{n}(\xi_{n}), n\geq 1\}$ *are WUOD* (*WLOD*) *with dominating coefficients* $g_{L}(n)(g_{U}(n))$, $n\geq1$.

(2) *If* ${\{\xi_{n}, n\geq1\}}$ *are nonnegative and WUOD with dominating coefficients* $g_{U}(n), n\geq1$, *then*, *for each* $n\geq1$,

$$ E\prod_{i=1}^{n} {\xi_{i}} \leq{g_{U}(n)}\prod_{i=1}^{n} E { \xi_{i}}. $$

*In particular*, *if* ${\{\xi_{n}, n\geq1\}}$ *are WUOD with dominating coefficients* $g_{U}(n), n\geq1$, *then*, *for each* $n\geq1$ *and any* $s>0$,

$$ E \exp \Biggl\{ s\prod_{i=1}^{n} { \xi_{i}} \Biggr\} \leq{g_{U}(n)}\prod _{i=1}^{n} E \exp\{s \xi_{i}\}. $$

## Main results

Now many studies of precise large deviations are focused on the dependent r.v.s. One can refer to Wang et al. [29], Liu [30], Tang [31], Liu [15], Yang and Wang [32], Wang et al. [20] and so on. Among them, Yang and Wang [32] consider the precise large deviations for extendedly negatively orthant dependent r.v.s, and Wang et al. [20] investigate the precise large deviations for WUOD and WLOD r.v.s. Their results have used the following assumptions.

### Assumption 1

For some $T>0$,

$$\begin{aligned} &0< {c_{1}}:=\liminf_{n\to\infty}{\inf_{x\geq T}} \frac{\sum_{i=1}^{n}\overline{F_{i}}(x)}{n{\overline{F}}(x)} \leq\limsup_{n\to\infty}{\sup_{x\geq T}} \frac{\sum_{i=1}^{n}\overline{F_{i}}(x)}{n{\overline{F}}(x)}=:{c_{2}}< \infty, \\ &0< {c_{3}}:=\liminf_{n\to\infty}{\inf _{x\geq T}}\frac{\sum_{i=1}^{n}F_{i}(-x)}{nF(-x)} \leq\limsup_{n\to\infty}{ \sup_{x\geq T}}\frac{\sum_{i=1}^{n} F_{i}(-x)}{nF(-x)}=:{c_{4}}< \infty. \end{aligned}$$

### Assumption 2

For all $i\geq1$, $F_{i}\in\mathscr{D}$. Furthermore, assume that for any $\varepsilon>0$, there exist some $w_{1}=w_{1}(\varepsilon)>1$ and $x_{1}=x_{1}(\varepsilon )>0$, irrespective of *i*, such that for all $i\geq1$, $1\leq w\leq w_{1}$ and $x\geq x_{1}$,

$$\frac{\overline{F_{i}}(wx)}{\overline{F_{i}}(x)}\geq L_{F_{i}}-\varepsilon, $$

or, equivalently, for any $\varepsilon>0$, there exist some $0< w_{2}=w_{2}(\varepsilon)<1$ and $x_{2}=x_{2}(\varepsilon)>0$, irrespective of *i*, such that for all $i\geq1$, $w_{2}\leq w\leq1$ and $x\geq x_{2}$,

$$\frac{\overline{F_{i}}(wx)}{\overline{F_{i}}(x)}\leq L_{F_{i}}^{-1}+\varepsilon. $$

### Assumption 3

For all $i\geq1$, $F_{i}\in\mathscr{D}$. Furthermore, assume that for any $0<\delta<1$, there exist some $v_{1}=v_{1}(\delta)>1$ and $x_{1}=x_{1}(\delta)>0$, irrespective of *i*, such that for all $i\geq1$, $1\leq v\leq v_{1}$ and $x\geq x_{1}$,

$$\frac{\overline{F_{i}}(vx)}{\overline{F_{i}}(x)}\geq\delta L_{F_{i}}, $$

or, equivalently, for any $\delta>1$, there exist some $0< v_{2}=v_{2}(\delta)<1$ and $x_{2}=x_{2}(\delta)>0$, irrespective of *i*, such that for all $i\geq1$, $v_{2}\leq v\leq1$ and $x\geq x_{2}$,

$$\frac{\overline{F_{i}}(vx)}{\overline{F_{i}}(x)}\leq\delta L_{F_{i}}^{-1}. $$

For the lower bound of the precise large deviations of the partial sums $S_{n}$, $n\geq1$, of the WOD r.v.s, when $\mu_{i}=0$, $i\geq1$, under Assumptions 1 and 3 and some other conditions, Theorem 2 of Wang et al. [20] obtained a lower bound: for every fixed $\gamma>0$,

$$ \liminf_{n\to\infty}{\inf_{x\geq\gamma n}}\frac {P(S_{n}>x)}{\sum_{i=1}^{n} L_{F_{i}}\overline{F_{i}}(x)} \geq1. $$

The following result will still consider the WOD r.v.s $X_{i}$ with finite means $\mu_{i}$, $i\geq1$, and only use Assumption 1 and some other conditions, without using Assumption 3, to obtain a lower bound of the precise large deviations of the partial sums $S_{n}$, $n\geq1$.

### Theorem 2.1

*Let* $\{X_{i}, i\geq1\}$ *be a sequence of WOD r*.*v*.*s with dominating coefficients* $g_{U}(n)\ (n\ge1)$ *and* $g_{L}(n)\ (n\ge1)$ *satisfying*, *for any* $\alpha\in(0,1)$,

2.1 $$ \lim_{n\to\infty}g_{U}(n) \bigl(n\overline{F}(n) \bigr)^{\alpha }=0, $$

*and for any* $\beta\in(0,1)$,

2.2 $$ \lim_{n\to\infty}g_{L}(n)n^{-\beta}=0. $$

*The distributions* $\{F_{i}, i\geq1\}$ *and* *F* *satisfy Assumption *1, $F\in\mathscr{D}$ *and*

2.3 $$ xF(-x)=o \bigl(\overline{F}(x) \bigr). $$

*Suppose that*, *for some* $r>1$,

$$ \sup_{i\geq1} E \bigl( (\mu_{i}-X_{i} )^{+} \bigr)^{r} < \infty. $$

*Then*, *for every fixed* $\gamma>0$,

2.4 $$ \liminf_{n\to\infty}{\inf_{x\geq\gamma n}}\frac{P (S_{n} - E(S_{n})>x )}{n{\overline{F}}(x)} \geq c_{1} L_{F}. $$

For the upper bound of the precise large deviations of the partial sums $S_{n}$, $n\geq1$, of the WUOD r.v.s, when $\mu_{i}=0$, $i\geq1$, under Assumptions 1 and 2 and some other conditions, Theorem 1 of Wang et al. [20] gave an upper bound: for every fixed $\gamma>0$,

$$ \limsup_{n\to\infty}{\sup_{x\geq\gamma n}}\frac {P(S_{n}>x)}{\sum_{i=1}^{n} L_{F_{i}}^{-1}\overline{F_{i}}(x)} \leq 1. $$

In the following result, we will use the following Assumption 4 to replace Assumption 2 and give an upper bound of the precise large deviations of the partial sums $S_{n}$, $n\geq1$, of the WUOD r.v.s. Assumption 4 is easier to verify than Assumption 2.

### Assumption 4

The expectations $\mu_{i}$, $i\geq1$, satisfy $\sum_{i=1}^{n} \mu_{i} =O(n)$.

Note that if $\sup_{i\geq1} \mu_{i} < \infty$ then Assumption 4 is satisfied. Particularly, the identically distributed random variables satisfy Assumption 4.

### Theorem 2.2

*Let* $\{X_{i}, i\geq1\}$ *be a sequence of WUOD r*.*v*.*s with dominating coefficients* $g_{U}(n)\ (n\ge1)$ *satisfying* (2.1). *The distributions* $\{F_{i}, i\geq1\}$ *and* *F* *satisfy Assumptions* 1 *and* 4 *and* $F\in\mathscr{D}$. *Then*, *for every fixed* $\gamma>0$,

2.5 $$ \limsup_{n\to\infty}{\sup_{x\geq\gamma n}}\frac{P (S_{n} - E(S_{n})>x )}{n{\overline{F}}(x)} \leq c_{2} {L_{F}} ^{-1}. $$

If we strengthen Assumption 1 to the following assumption, Assumption 4 can be dropped in Theorem 2.2.

### Assumption 1^∗^

$$\begin{aligned} &0< {c_{5}}:=\liminf_{n\to\infty}{\inf _{x\geq0}}\frac{\sum_{i=1}^{n}\overline{F_{i}}(x)}{n{\overline{F}}(x)} \leq\limsup_{n\to\infty}{ \sup_{x\geq0}}\frac{\sum_{i=1}^{n}\overline{F_{i}}(x)}{n{\overline{F}}(x)}=:{c_{6}}< \infty, \\ &0< {c_{7}}:=\liminf_{n\to\infty}{\inf _{x\geq0}}\frac{\sum_{i=1}^{n}F_{i}(-x)}{nF(-x)} \leq\limsup_{n\to\infty}{ \sup_{x\geq0}}\frac{\sum_{i=1}^{n} F_{i}(-x)}{nF(-x)}=:{c_{8}}< \infty. \end{aligned}$$

### Theorem 2.3

*Let* $\{X_{i}, i\geq1\}$ *be a sequence of WUOD r*.*v*.*s with dominating coefficients* $g_{U}(n)\ (n\ge1)$ *satisfying* (2.1). *The distributions* $\{F_{i}, i\geq1\}$ *and* *F* *satisfy Assumptions* 1^∗^ *and* $F\in\mathscr{D}$. *Then*, *for every fixed* $\gamma>0$,

$$ \limsup_{n\to\infty}{\sup_{x\geq\gamma n}}\frac{P (S_{n} - E(S_{n})>x )}{n{\overline{F}}(x)} \leq c_{6} {L_{F}} ^{-1}. $$

When $\{X_{i}, i\geq1\}$ are independent but non-identically distributed r.v.s, then $g_{U}(n)=g_{L}(n)\equiv1, n\geq1$, and (2.1) and (2.2) hold. By Theorems 2.1 and 2.2, the following two corollaries can be obtained.

### Corollary 2.1

*Let* $\{X_{i}, i\geq1\}$ *be a sequence of independent but non*-*identically distributed r*.*v*.*s*. *The distributions* $\{F_{i}, i\geq1\}$ *and* *F* *satisfy Assumption *1, $F\in\mathscr{C}$ *and* (2.3). *Suppose that*, *for some* $r>1$, $\sup_{i\geq1} E( (\mu_{i}-X_{i} )^{+})^{r} <\infty. \nonumber $ *Then*, *for every fixed* $\gamma>0$,

2.6 $$ \liminf_{n\to\infty}{\inf_{x\geq\gamma n}}\frac{P (S_{n} - E(S_{n})>x )}{n{\overline{F}}(x)} \geq c_{1}. $$

### Corollary 2.2

*Let* $\{X_{i}, i\geq1\}$ *be a sequence of independent but non*-*identically distributed r*.*v*.*s*. *The distributions* $\{F_{i}, i\geq1\}$ *and* *F* *satisfy Assumptions* 1 *and* 4 *and* $F\in \mathscr{C}$. *Then*, *for every fixed* $\gamma>0$,

2.7 $$ \limsup_{n\to\infty}{\sup_{x\geq\gamma n}}\frac{P (S_{n} - E(S_{n})>x )}{n{\overline{F}}(x)} \leq c_{2}. $$

### Remark 2.1

In Theorem of Paulauskas and Skučaitė [10], the case that $\{X_{i}, i\geq1\}$ is a sequence of independent but non-identically distributed r.v.s was also considered, and the following result was obtained.

Let $\{X_{i}, i\geq1\}$ be a sequence of independent but non-identically distributed r.v.s. Assume that:

1. $\mu_{i}=0$;
2. $F\in\mathscr{R}_{-\alpha}$ for $\alpha>1$ and the distributions $\{F_{i}, i\geq1\}$ and *F* satisfy Assumption 1 for $c_{i}\equiv1, i=1,2,3,4$.
3. There exists a sequence of constants $a_{n}$ such that $a_{n}\uparrow \infty$, $\sup a_{n} n^{-1}<\infty$ and $\sum_{n=1}^{\infty}a_{n}^{-p}E \vert X_{n} \vert ^{p}<\infty$ for some $1< p\leq2$.

Then

2.8 $$ \lim_{n\to\infty}\frac{P (S_{n}>t_{n} )}{n{\overline{F}}(t_{n})}=1 $$

for all sequences $t_{n}\in(-\infty, \infty)$ satisfying the conditions $\limsup_{n\to\infty}nt_{n}^{-1}<\infty$ and $\overline {F_{i}}(t_{n})=o(n\overline{F}(t_{n})), i\geq1$.

From the proof of Theorem of Paulauskas and Skučaitė [10], we note that $t_{n}$ should be positive.

If conditions (1) and (2) hold, then the conditions of Corollary 2.2 are satisfied. If we let $a=\lim_{n\to\infty} \sup nt_{n}^{-1}$, then $a\in(0,\infty)$ and $t_{n}\geq(a+1)^{-1}n$ for large *n*. Thus, it follows from (2.7) that

$$\limsup_{n\to\infty}\frac{P (S_{n}>t_{n} )}{n{\overline{F}}(t_{n})}\leq1, $$

which means that the upper bound of $P(S_{n}>t_{n})$ in (2.8) can be obtained from Corollary 2.2.

Comparing Corollary 2.1 and Theorem of Paulauskas and Skučaitė [10], it can be found that they give the lower bound of the precise large deviations of $S_{n}, n\geq1$, under different conditions.

When $\{X_{i}, i\geq1\}$ and *X* are identically distributed r.v.s, Assumptions 1 and 1^∗^ are satisfied. The following two corollaries can be obtained directly from Theorems 2.1 and 2.3.

### Corollary 2.3

*Let* $\{X_{i}, i\geq1, X\}$ *be identically distributed r*.*v*.*s with common distribution* $F\in\mathscr{D}$. *Assume that* $\{X_{i}, i\geq1\} $ *are WOD r*.*v*.*s with dominating coefficients* $g_{U}(n)\ (n\ge1)$ *and* $g_{L}(n)\ (n\ge1)$ *satisfying* (2.1) *and* (2.2). *If* $E(X^{-})^{r}<\infty$ *for some* $r>1$ *and relation* (2.3) *holds*, *then for every fixed* $\gamma>0$,

$$ \liminf_{n\to\infty}{\inf_{x\geq\gamma n}}\frac {P(S_{n}-E(S_{n})>x)}{n \overline{F}(x)} \geq L_{F}. $$

### Corollary 2.4

*Let* $\{X_{i}, i\geq1, X\}$ *be identically distributed r*.*v*.*s with common distribution* $F\in\mathscr{D}$. *Assume that* $\{X_{i}, i\geq1\} $ *are WUOD r*.*v*.*s with dominating coefficients* $g_{U}(n)\ (n\ge1)$ *satisfying* (2.1). *Then*, *for every fixed* $\gamma>0$,

$$ \limsup_{n\to\infty}{\sup_{x\geq\gamma n}}\frac {P(S_{n}-E(S_{n})>x)}{n \overline{F}(x)} \leq L_{F}^{-1}. $$

### Remark 2.2

(1) We note that, for any fixed $d>0$ and $r>1$,

2.9 $$\begin{aligned} E \bigl( (d-X )^{+} \bigr)^{r} < \infty\quad\Leftrightarrow\quad E \bigl(X^{-} \bigr)^{r}< \infty. \end{aligned}$$

In fact, on the one hand,

$$\begin{aligned} E \bigl( (d-X )^{+} \bigr)^{r} &= E(d-X)^{r}{ \mathbf {1}}_{\{0\leq X\leq d\}} +E(d-X)^{r}{\mathbf {1}}_{\{X< 0\}} \\ &\geq E \bigl(X^{-} \bigr)^{r}. \end{aligned}$$

On the other hand, by $C_{r}$-inequality, we have

$$\begin{aligned} E \bigl( (d-X )^{+} \bigr)^{r}\leq E \bigl(d+X^{-} \bigr)^{r} \leq 2^{r-1} \bigl(d^{r}+E \bigl(X^{-} \bigr)^{r} \bigr). \end{aligned}$$

Thus (2.9) can be obtained.

(2) Corollaries 1 and 2 of Wang et al. [20] consider the identically distributed r.v.s $X_{i}\ (i\geq1)$ with finite mean $\mu_{i}=0\ (i\geq1)$. Corollaries 2.3 and 2.4 deal with the case that $\mu_{i}\neq0$ ($i\geq1$). We note that when $\mu_{i}\neq0$, $i\geq 1$, Corollaries 2.3 and 2.4 cannot be obtained directly from Corollaries 1 and 2 of Wang et al. [20].

## Proofs of results

### Some lemmas

Before proving the main results, we first give some lemmas. The following lemma is a combination of Proposition 2.2.1 of Bingham et al. [1] and Lemma 3.5 of Tang and Tsitsiashvili [33].

#### Lemma 3.1

*If* $V\in\mathscr{D}$, *then*

(1) *for each* $\rho>J_{V}^{+}$, *there exist positive constants* *A* *and* *B* *such that the inequality*

$$ \frac{\overline{V}(y)}{\overline{V}(x)}\leq A \biggl(\frac {x}{y} \biggr)^{\rho} $$

*holds for all* $x\geq y\geq B$;

(2) *it holds for each* $\rho>J_{V}^{+}$ *that*

$$ x^{-\rho}=o \bigl(\overline{V}(x) \bigr). $$

#### Lemma 3.2

*Let* $\{\xi_{k}, k\geq1\}$ *be WUOD r*.*v*.*s with dominating coefficients* $g_{U}(n)$ $(n\ge1)$, *with distributions* $\{V_{k}, k\geq1\}$ *and finite mean* 0, *satisfying* $\sup_{k\geq1}E(\xi_{k}^{+})^{r}<\infty$ *for some* $r>1$. *Then*, *for each fixed* $\gamma>0$ *and* $p>0$, *there exist positive numbers* *v* *and* $C=C(v, \gamma)$, *irrespective of* *x* *and* *n*, *such that for all* $n=1, 2, \ldots$ *and* $x\geq\gamma n$,

$$ P \Biggl(\sum_{k=1}^{n} { \xi_{k}}\geq x \Biggr) \leq{\sum_{k=1}^{n} {\overline{V_{k}}(vx)}}+{C g_{U}(n)x^{-p}}. $$

This lemma extends Lemma 2.3 of Tang [31] to the WUOD r.v.s with different distributions. The proof is similar to the proof of Lemma 2.3 of Tang [31]. However, for the completeness of the proof, we give the following proof with some modifications.

#### Proof

If we prove the result is correct for all $n> n_{0}$ and $x\geq\gamma n$, where $n_{0}$ is a positive integer, then using the inequality

3.1 $$\begin{aligned} P \Biggl(\sum_{k=1}^{n} { \xi_{k}}\geq x \Biggr) &\leq{\sum_{k=1}^{n} P \biggl(\xi_{k}\geq\frac{x}{n_{0}} \biggr)} \\ &= \sum_{k=1}^{n} \overline{V_{k}} \biggl(\frac{x}{n_{0}} \biggr), \quad n=1, 2, \ldots, n_{0}, \end{aligned}$$

the result can be extended to all $n=1, 2, \ldots$ .

For any fixed $v>0$, we denote $\widetilde{\xi_{k}}=\min\{\xi_{k}, vx\} $, $k=1, 2, \ldots$ , by Proposition 1.1(1), they are still WUOD with dominating coefficients $g_{U}(n)$, $n\ge1$. Using a standard truncation argument, we get

3.2 $$\begin{aligned} P \Biggl(\sum_{k=1}^{n} { \xi_{k}}\geq x \Biggr) &=P \Biggl(\sum_{k=1}^{n} {\xi_{k}}\geq x, \max_{1\leq k\leq n} \xi_{k} > vx \Biggr)+P \Biggl(\sum_{k=1}^{n} { \xi_{k}}\geq x, \max_{1\leq k\leq n} \xi_{k} \leq vx \Biggr) \\ &\leq\sum_{k=1}^{n} \overline{V_{k}}(vx)+P \Biggl(\sum_{k=1}^{n} {\widetilde{ \xi_{k}}}\geq x \Biggr). \end{aligned}$$

Now, we estimate the second term in (3.2). For a positive number *h*, which we shall specify later, using Chebyshev’s inequality and Proposition 1.1(2), we have

3.3 $$ P \Biggl(\sum_{k=1}^{n} {\widetilde{ \xi_{k}}}\geq x \Biggr)\leq g_{U}(n)e^{-hx}\prod _{i=1}^{n} E e^{h \widetilde{\xi_{k}}}. $$

For some $1< q<\min\{r,2\}$, $E e^{h \widetilde{\xi_{k}}}$ is bounded from above by

3.4 $$ \int_{-\infty}^{0} \bigl(e^{hu}-1 \bigr)V_{k}(du)+ \int_{0}^{vx}\frac {e^{hu}-1-hu}{u^{q}} u^{q} V_{k}(du)+ \bigl(e^{hvx}-1 \bigr) \overline{V_{k}}(vx)+ {h u_{k}^{+}} +1, $$

here $u_{k}^{+} =E \xi_{k} \mathbf{1}_{\{\xi_{k}>0\}}$. For the first term in (3.4), since

$$ 0\leq\frac{e^{hu}-1-hu}{h}\leq u \bigl(e^{hu}-1 \bigr)\leq{-u}\quad \mbox{for all } u \leq0, $$

by the dominated convergence theorem, we have

$$ \lim_{h\searrow0}\frac{\int_{-\infty }^{0}(e^{hu}-1)V_{k}(du)}{h}=\lim_{h\searrow0} \int_{-\infty }^{0}\frac{e^{hu}-1-hu}{h}V_{k}(du)+ {u_{k}^{-}}= {u_{k}^{-}}, $$

where $u_{k}^{-} =E \xi_{k} \textbf{1}_{\{\xi_{k}\leq0\}}$. Hence, there exists a real function $\alpha(\cdot)$ with $\alpha(h)\rightarrow0$ as $h\searrow0$ such that

3.5 $$ \int_{-\infty}^{0} \bigl(e^{hu}-1 \bigr)V_{k}(du)= \bigl(1+ \alpha(h) \bigr)h u_{k}^{-}. $$

By the monotonicity of $(e^{hu}-1-hu)/{u^{q}}$ for $u\in(0,\infty)$, the second term in (3.4) is bounded by

3.6 $$ \frac{e^{hvx}-1-hvx}{(vx)^{q}}E \bigl(\xi_{k}^{+} \bigr)^{q}, $$

where $\xi_{k}^{+} = \max\{\xi_{k},0\}$. Applying (3.5) and (3.6) to (3.4), from (3.3) we obtain that

3.7 $$\begin{aligned} &P \Biggl(\sum_{k=1}^{n}{\widetilde{ \xi_{k}}}\geq x \Biggr) \\ &\quad\leq g_{U}(n)e^{-hx}\prod _{k=1}^{n} \biggl\{ \bigl(1+ \alpha(h) \bigr)h u_{k}^{-} +\frac {e^{hvx}-1-hvx}{(vx)^{q}}E \bigl(\xi_{k}^{+} \bigr)^{q} \\ &\qquad{} + \bigl(e^{hvx}-1 \bigr)\overline{V_{k}}(vx)+ h u_{k}^{+} +1 \biggr\} \\ &\quad\leq g_{U}(n)e^{-hx}\prod_{k=1}^{n} \exp \biggl\{ \bigl(1+ \alpha(h) \bigr)h u_{k}^{-} +\frac{e^{hvx}-1}{(vx)^{q}}E \bigl(\xi_{k}^{+} \bigr)^{q} + \bigl(e^{hvx}-1 \bigr) \overline {V_{k}}(vx)+ h u_{k}^{+} \biggr\} \\ &\quad=g_{U}(n)\exp \Biggl\{ \sum_{k=1}^{n} \alpha(h) h u_{k}^{-} +\frac {e^{hvx}-1}{(vx)^{q}}\sum _{k=1}^{n}E \bigl(\xi_{k}^{+} \bigr)^{q} + \bigl(e^{hvx}-1 \bigr)\sum _{k=1}^{n}\overline{V_{k}}(vx)- hx \Biggr\} , \end{aligned}$$

where at the second step we use the inequality $s+1\leq e^{s}$ for all *s*. In (3.7), take

$$h=\frac{1}{vx}\log \biggl(\frac{v^{q-1}x^{q}}{\sum_{k=1}^{n}E(\xi _{k}^{+})^{q}}+1 \biggr). $$

Since $\sup_{k\geq1} E(\xi_{k})^{r}<\infty$, there exists a constant $M_{1}>0$, irrespective of *x* and *n*, such that for all $n=1,2,\ldots$ ,

$$ \Biggl\vert \sum_{k=1}^{n} u_{k}^{-} \Biggr\vert = \sum_{k=1}^{n} u_{k}^{+} \leq n M_{1}. $$

By some calculation, we know that, for all large *n* such that for all $x\geq\gamma n$,

$$ \bigl\vert \alpha(h)M_{1} \bigr\vert \leq\frac{1}{2}\gamma. $$

Then, for all large *n* and $x\geq\gamma n$,

$$ \Biggl\vert \sum_{k=1}^{n} \alpha(h)u_{k}^{-} \Biggr\vert \leq\frac{1}{2}\gamma n \leq \frac{x}{2}. $$

Therefore, for all large *n* and $x\geq\gamma n$, the right-hand side of (3.7) is bounded from above by

3.8 $$\begin{aligned} &g_{U}(n)\exp \biggl\{ \frac{1}{2v}\log \biggl( \frac{v^{q-1}x^{q}}{\sum_{k=1}^{n}E(\xi_{k}^{+})^{q}}+1 \biggr)+ \frac{1}{v}+\frac{v^{q-1}x^{q}\sum_{k=1}^{n}\overline{V_{k}}(vx)}{\sum_{k=1}^{n}E(\xi_{k}^{+})^{q}} \\ &\qquad{} - \frac{1}{v}\log \biggl(\frac{v^{q-1}x^{q}}{\sum_{k=1}^{n}E(\xi_{k}^{+})^{q}}+1 \biggr) \biggr\} \\ &\quad\leq g_{U}(n)\exp \biggl\{ \frac{1}{v}+\frac{v^{q-1}x^{q}\sum_{k=1}^{n}\overline{V_{k}}(vx)}{\sum_{k=1}^{n}E(\xi_{k}^{+})^{q}} \biggr\} \biggl(\frac{v^{q-1}x^{q}}{\sum_{k=1}^{n}E(\xi_{k}^{+})^{q}} \biggr)^{-\frac {1}{2v}} \\ &\quad= g_{U}(n)\exp \biggl\{ \frac{1}{v}+\frac{v^{q-1}x^{q}\sum_{k=1}^{n}\overline{V_{k}}(vx)}{\sum_{k=1}^{n}E(\xi_{k}^{+})^{q}} \biggr\} \biggl(\frac{v^{q-1}x}{\sum_{k=1}^{n}E(\xi_{k}^{+})^{q}} \biggr)^{-\frac {1}{2v}} x^{-\frac{q-1}{2v}} \\ &\quad\leq g_{U}(n)\exp \biggl\{ \frac{1}{v}+\frac{v^{q-1}x^{q}\sum_{k=1}^{n}\overline{V_{k}}(vx)}{\sum_{k=1}^{n}E(\xi_{k}^{+})^{q}} \biggr\} \biggl(\frac{v^{q-1}\gamma}{\max_{k\geq1}E(\xi _{k}^{+})^{q}} \biggr)^{-\frac{1}{2v}} x^{-\frac{q-1}{2v}}. \end{aligned}$$

Since, for each $k=1,2,\ldots$ and for all $x>0$,

$$ E \bigl(\xi_{k}^{+} \bigr)^{q}\geq \int_{x}^{\infty}u^{q} V_{k}(du) \geq x^{q} \overline {V_{k}}(x), $$

then the right-hand side of (3.8) is bounded from above by

$$\begin{aligned} g_{U}(n)e^{\frac{2}{v}} \biggl(\frac{v^{q-1}\gamma}{\max_{k\geq 1}E(\xi_{k}^{+})^{q}} \biggr)^{-\frac{1}{2v}} x^{-\frac {q-1}{2v}}=:C g_{U}(n) x^{-\frac{q-1}{2v}} \leq C g_{U}(n) x^{-p}, \end{aligned}$$

where $C=e^{\frac{2}{v}} (\frac{v^{q-1}\gamma}{\max_{k\geq 1}E(\xi_{k}^{+})^{q}} )^{-\frac{1}{2v}}<\infty$. In the last step, we take a proper $v>0$ such that $\frac{q-1}{2v}>p$. This completes the proof of this lemma. □

#### Lemma 3.3

*Assumption *1^∗^ *implies Assumptions* 1 *and* 4.

#### Proof

It is clear that Assumption 1^∗^ implies Assumption 1. Now we prove Assumption 1^∗^ implies Assumption 4. For sufficiently large *n*, by Assumption 1^∗^, we have

$$\begin{aligned} \sum_{i=1}^{n} \vert \mu_{i} \vert &\leq\sum_{i-1}^{n} \int_{0}^{\infty}\bigl(\overline {F_{i}}(y)+F_{i}(-y) \bigr)\,dy \\ &\leq 2n \int_{0}^{\infty}\bigl(c_{6} \overline{F}(y)+c_{8}F(-y) \bigr)\,dy< \infty, \end{aligned}$$

which means that Assumption 4 holds. This completes the proof of this lemma. □

### Proof of Theorem 2.1

We use the line of proof of Theorem 3.1 in Ng et al. [9] to prove this result. For any $\lambda>1$,

3.9 $$\begin{aligned} &P \bigl(S_{n} - E(S_{n})>x \bigr) \\ &\quad\geq P \Biggl(S_{n} - \sum_{k=1}^{n} \mu_{k} >x, \bigcup_{j=1}^{n}\{ X_{j}>\lambda x\} \Biggr) \\ &\quad\geq\sum_{j=1}^{n}P \Biggl(S_{n} - \sum_{k=1}^{n} \mu_{k} >x, X_{j}>\lambda x \Biggr)-\sum _{1\leq j< l\leq n}P \Biggl(S_{n} - \sum _{k=1}^{n}\mu_{k} >x, X_{j}> \lambda x, X_{l}>\lambda x \Biggr) \\ &\quad\geq\sum_{j=1}^{n}P \Biggl(S_{n} - \sum_{k=1}^{n} \mu_{k} >x, X_{j}>\lambda x \Biggr)-\sum _{1\leq j< l\leq n}P (X_{j}>\lambda x, X_{l}>\lambda x ) \\ &\quad\geq\sum_{j=1}^{n}P \Biggl(S_{n} - X_{j}-\sum_{k=1}^{n} \mu_{k} >x-\lambda x, X_{j}>\lambda x \Biggr)-g_{U}(n) \Biggl(\sum_{j=1}^{n}\overline {F_{j}}(\lambda x) \Biggr)^{2} \\ &\quad\geq\sum_{j=1}^{n}\overline{F_{j}}( \lambda x)- \sum_{j=1}^{n}P \Biggl(S_{n}^{(j)}-\sum_{k=1}^{n} \mu_{k} \leq(1-\lambda) x \Biggr)-g_{U}(n) \Biggl(\sum _{j=1}^{n}\overline{F_{j}}( \lambda x) \Biggr)^{2} \\ &\quad=\sum_{j=1}^{n}\overline{F_{j}}( \lambda x) \Biggl(1-g_{U}(n)\sum_{j=1}^{n} \overline{F_{j}}(\lambda x) \Biggr)-\sum_{j=1}^{n}P \Biggl(S_{n}^{(j)}-\sum_{k=1}^{n} \mu_{k} \leq(1-\lambda) x \Biggr) \\ &\quad=:I_{1}-I_{2}, \end{aligned}$$

where $S_{n}^{(j)}=\sum_{1\leq k\neq j\leq n}X_{k}$. In the fourth step, the definition of WUOD was used, and we use an elementary inequality $P(AB)\geq P(B)-P(A^{c})$ for all events *A* and *B* in the fifth step.

We estimate the second term in (3.9). For all large *n* and $x\geq\gamma n$, we have

$$\begin{aligned} I_{2} \leq\sum_{j=1}^{n} P \biggl(\sum_{1\leq k\neq j\leq n}(\mu_{k}-X_{k}) \geq\frac{(\lambda-1)x}{2} \biggr). \end{aligned}$$

By Proposition 1.1(1), the r.v.s $\{\mu_{k}-X_{k}, k\geq1\}$ are WUOD with dominating coefficients $g_{L}(n)$, $n\ge1$. Then, for arbitrarily fixed $\gamma>0$ and $\beta\in(0,1)$, by Lemma 3.2, there exist positive constants $v_{0}$ and *C*, irrespective of *x* and *n*, such that

$$\begin{aligned} & \sum_{j=1}^{n} P \biggl(\sum _{1\leq k\neq j\leq n}(\mu _{k}-X_{k})\geq \frac{(\lambda-1)x}{2} \biggr) \\ &\quad\leq\sum_{j=1}^{n}\sum _{1\leq k\neq j\leq n} P \biggl(\mu _{k}-X_{k}\geq \frac{(\lambda-1)x}{2v_{0}} \biggr) + Cng_{L}(n)x^{-(\beta+ J_{F}^{+})} \\ &\quad\leq n \sum_{k=1}^{n}F_{k} \biggl(\frac{-(\lambda-1)x}{4v_{0}} \biggr)+ Cng_{L}(n)x^{-(\beta+ J_{F}^{+})} \end{aligned}$$

holds for all large *n* and $x\geq\gamma n$. By Lemma 3.1(2), $F\in\mathscr{D}$ and (2.2), for all large *n* and $x\geq\gamma n$,

$$\begin{aligned} ng_{L}(n)x^{-(\beta+ J_{F}^{+})}&= ng_{L}(n)x^{-\frac{\beta }{2}}x^{-(\frac{\beta}{2}+J_{F}^{+})} \\ &\leq ng_{L}(n) n^{-\frac{\beta}{2}} {\gamma}^{-\frac{\beta }{2}}x^{-(\frac{\beta}{2}+J_{F}^{+})} \\ &=o \bigl(n \overline{F}(\lambda x) \bigr). \end{aligned}$$

By Assumption 1, $F\in\mathscr{D}$ and (2.3),

$$\begin{aligned} &\limsup_{n\to\infty}{\sup_{x\geq\gamma n}}\frac{\sum_{k=1}^{n}F_{k} (\frac{-(\lambda-1)x}{4v_{0}})}{\overline{F}(\lambda x)} \\ &\quad\leq\limsup_{n\to\infty}{\sup_{x\geq\gamma n}} \frac {\sum_{k=1}^{n}F_{k} (\frac{-(\lambda-1)x}{4v_{0}})}{nF(\frac{-(\lambda -1)x}{4v_{0}})}{\gamma}^{-1} \limsup_{x\to\infty} \frac{xF(\frac{-(\lambda -1)x}{4v_{0}})}{\overline{F}(\frac{(\lambda-1)x}{4v_{0}})} \limsup_{x\to\infty}\frac{\overline{F}(\frac{(\lambda -1)x}{4v_{0}})}{{\overline{F}(\lambda x)}} \\ &\quad=0. \end{aligned}$$

Therefore, it holds that

3.10 $$ \limsup_{n\to\infty}{\sup_{x\geq\gamma n}} \frac {I_{2}}{n\overline{F}(\lambda x)}=0. $$

Now we estimate $I_{1}$. By (2.1), $F\in\mathscr{D}$ and Assumption 1,

$$\begin{aligned} &\limsup_{n\to\infty}{\sup_{x\geq\gamma n}}g_{U}(n) \sum_{j=1}^{n}\overline{F_{j}}( \lambda x) \\ &\quad\leq\limsup_{n\to\infty}{\sup_{x\geq\gamma n}}g_{U}(n)n \overline{F}(n)\frac{\overline{F}(\lambda\gamma n)}{\overline{F}(n)}\frac{\sum_{j=1}^{n}\overline{F_{j}}(\lambda x)}{n\overline{F}(\lambda x)} \\ &\quad\leq\limsup_{n\to\infty}g_{U}(n)n\overline{F}(n)\limsup _{n\to\infty}\frac{\overline{F}(\lambda\gamma n)}{\overline{F}(n)}\limsup_{n\to\infty}{\sup _{x\geq \gamma n}}\frac{\sum_{j=1}^{n}\overline{F_{j}}(\lambda x)}{n\overline {F}(\lambda x)} \\ &\quad=0. \end{aligned}$$

By Assumption 1, we have that

3.11 $$ \liminf_{n\to\infty}{\inf_{x\geq\gamma n}}\frac {I_{1}}{n\overline{F}(\lambda x)} \geq\liminf_{n\to\infty }{\inf_{x\geq\gamma n}} \frac{\sum_{j=1}^{n}\overline{F_{j}}(\lambda x)}{n\overline{F}(\lambda x)}\geq c_{1}. $$

Thus, by (3.9)-(3.11),

$$ \liminf_{n\to\infty}{\inf_{x\geq\gamma n}}\frac{P (S_{n} - E(S_{n})>x )}{n{\overline{F}}(x)} \geq c_{1}\lim_{\lambda\searrow1}\liminf_{x\to\infty} \frac {\overline{F}(\lambda x)}{\overline{F}(x)}= c_{1} L_{F}. $$

The proof of Theorem 2.1 is completed.

### Proof of Theorem 2.2

For any fixed positive integer *m* and for any $\theta\in (0,\frac{m}{m+1} )$, we define $\widetilde{X_{k}}:=\min\{X_{k}, \theta x\}$, $k\geq1$, $\widetilde {S_{n}}:=\sum_{k=1}^{n}\widetilde{X_{k}}$, $n\geq1$ and $\widetilde{x_{n}}:= x+ \sum_{k=1}^{n}\mu_{k}$, $n\geq1$. By a standard truncation argument, we have

3.12 $$\begin{aligned} &P \bigl(S_{n} - E(S_{n})>x \bigr) \\ &\quad=P \Biggl(S_{n} - \sum_{k=1}^{n} \mu_{k} >x, \max_{1\leq k\leq n}X_{k}>\theta x \Biggr)+ P \Biggl(S_{n} - \sum_{k=1}^{n} \mu_{k} >x, \max_{1\leq k\leq n}X_{k} \leq\theta x \Biggr) \\ &\quad\leq\sum_{k=1}^{n}P(X_{k}> \theta x) +P(\widetilde{S_{n}}>\widetilde {x_{n}}). \end{aligned}$$

We estimate the second term in (3.12). Let $a=\max\{-m^{-1}\log (n\overline{F}(\theta x)),1\}$, which tends to ∞ uniformly for $x\geq\gamma n$ when *n* tends to ∞. For any fixed $h=h(x,n)>0$, when *n* is sufficiently large, we have

3.13 $$\begin{aligned} &\frac{P(\widetilde{S_{n}}>\widetilde{x_{n}})}{n\overline{F}(\theta x)} \\ &\quad\leq g_{U}(n)e^{ma-h\widetilde{x_{n}}}\prod_{k=1}^{n}E e^{h\widetilde {X_{k}}} \\ &\quad=g_{U}(n)e^{ma-h\widetilde{x_{n}}}\prod_{k=1}^{n} \biggl\{ \int_{-\infty }^{\theta x} \bigl(e^{hy}-1 \bigr)F_{k}(dy)+ \bigl(e^{h\theta x}-1 \bigr)\overline{F_{k}}( \theta x)+1 \biggr\} \\ &\quad\leq g_{U}(n)\exp \Biggl\{ ma-h\widetilde{x_{n}}+\sum _{k=1}^{n} \int _{-\infty}^{\theta x} \bigl(e^{hy}-1 \bigr)F_{k}(dy)+ \bigl(e^{h\theta x}-1 \bigr)\sum _{k=1}^{n}\overline{F_{k}}(\theta x) \Biggr\} \\ &\quad\leq g_{U}(n)\exp \Biggl\{ ma-h\widetilde{x_{n}}+\sum _{k=1}^{n} \biggl(e^{\frac{h\theta x}{a^{2}}} \int_{-\infty}^{\frac{\theta x}{a^{2}}}htF_{k}(dy)+e^{h \theta x} \overline{F_{k}} \biggl(\frac{\theta x}{a^{2}} \biggr) \biggr) \\ &\qquad{}+ \bigl(e^{h\theta x}-1 \bigr)\sum_{k=1}^{n} \overline {F_{k}}(\theta x) \Biggr\} \\ &\quad\leq g_{U}(n)\exp \Biggl\{ ma-h\widetilde{x_{n}}+e^{\frac{h\theta x}{a^{2}}}h \sum_{k=1}^{n}\mu_{k}+e^{h \theta x} \sum_{k=1}^{n}\overline {F_{k}} \biggl(\frac{\theta x}{a^{2}} \biggr)+ \bigl(e^{h\theta x}-1 \bigr)\sum _{k=1}^{n}\overline {F_{k}}(\theta x) \Biggr\} \\ &\quad=g_{U}(n)\exp \Biggl\{ ma-hx + h \bigl(e^{\frac{h\theta x}{a^{2}}}-1 \bigr)\sum _{k=1}^{n}\mu_{k} +e^{h \theta x}\sum_{k=1}^{n} \overline{F_{k}} \biggl(\frac{\theta x}{a^{2}} \biggr) \\ &\qquad{}+ \bigl(e^{h\theta x}-1 \bigr)\sum_{k=1}^{n} \overline{F_{k}}(\theta x) \Biggr\} . \end{aligned}$$

For $\rho> J_{F}^{+}$, take $h=\frac{ma-2\rho\log{a}}{\theta x}$. By Assumption 4, for all large *n* and $x\geq\gamma n$, it holds that

3.14 $$\begin{aligned} h \bigl(e^{\frac{h\theta x}{a^{2}}}-1 \bigr)\sum_{k=1}^{n} \mu_{k} &=h \bigl(e^{\frac{m}{a}}e^{-\rho\frac{\log{a^{2}}}{a^{2}}}-1 \bigr)\sum _{k=1}^{n}\mu_{k} \\ &= o(1) h n \\ &=o(hx). \end{aligned}$$

For any $\delta_{1}>0$, by Assumption 1 and Lemma 3.1(1), for all large *n* and $x\geq\gamma n$, it holds that

3.15 $$\begin{aligned} e^{h \theta x}\sum_{k=1}^{n} \overline{F_{k}} \biggl(\frac{\theta x}{a^{2}} \biggr)&\leq e^{h \theta x} n \overline{F} \biggl(\frac{\theta x}{a^{2}} \biggr) (c_{2}+\delta_{1}) \\ &\leq e^{h \theta x} n A a^{2\rho}\overline{F}(\theta x) (c_{2}+\delta _{1}) \\ &=A(c_{2}+\delta_{1}). \end{aligned}$$

For the above $\delta_{1}>0$, by Assumption 1, for all large *n* and $x\geq\gamma n$, it holds that

3.16 $$\begin{aligned} \bigl(e^{h \theta x}-1 \bigr)\sum_{k=1}^{n} \overline{F_{k}}(\theta x)&\leq \bigl(e^{h \theta x}-1 \bigr) n \overline{F}(\theta x) (c_{2}+\delta_{1}) \\ &= \bigl(a^{-2\rho}-e^{-ma} \bigr) (c_{2}+ \delta_{1}) \\ &=o(1). \end{aligned}$$

Hence, by (3.13)-(3.16), for all large *n* and $x\geq \gamma n$,

$$\begin{aligned} \frac{P(\widetilde{S_{n}}>\widetilde{x_{n}})}{n\overline{F}(\theta x)} &\leq g_{U}(n)e^{-a}\exp \bigl\{ ma-hx+o(hx)+A(c_{2}+\delta_{1})+o(1)+a \bigr\} \\ &= g_{U}(n) \bigl(n\overline{F}(\theta x) \bigr)^{\frac{1}{m}}\exp \biggl\{ a \biggl(m+1-\frac{m}{\theta}+o(1) \biggr) +A(c_{2}+ \delta_{1})+o(1) \biggr\} . \end{aligned}$$

By (2.1) and $F\in\mathscr{D}$,

$$ \limsup_{n\to\infty}{\sup_{x\geq\gamma n}}g_{U}(n) \bigl(n\overline{F}(\theta x) \bigr)^{\frac{1}{m}}=0. $$

Since $m+1-\frac{m}{\theta}<0$ and $\liminf_{n\to\infty }{\inf_{x\geq\gamma n}}a=\infty$, it holds that

$$ \limsup_{n\to\infty}{\sup_{x\geq\gamma n}}\frac {P(\widetilde{S_{n}}>\widetilde{x_{n}})}{n\overline{F}(\theta x)} =0. $$

Hence, by (3.12),

3.17 $$ \limsup_{n\to\infty}{\sup_{x\geq\gamma n}}\frac {P({S_{n}}-E(S_{n})>x)}{n\overline{F}(\theta x)} \leq\limsup_{n\to\infty}{\sup_{x\geq\gamma n}} \frac{\sum_{k=1}^{n}\overline{F_{k}}(\theta x)}{n\overline{F}(\theta x)}\leq c_{2}. $$

By the definition of $L_{F}$, for any $\varepsilon>0$, there exist $x_{0}>0$ and $0<\theta_{0}<1$ such that for all $x\geq x_{0}$ and $\theta _{0}\leq\theta\leq1$,

3.18 $$ \frac{\overline{F}(\theta x)}{\overline{F}(x)}\leq L_{F}^{-1} + \varepsilon. $$

Take a positive integer *m* such that $\theta\leq\frac{m}{m+1}$. Then, for all $\theta_{0}\leq\theta\leq\frac{m}{m+1}$, by (3.17) and (3.18),

$$\begin{aligned} \limsup_{n\to\infty}{\sup_{x\geq\gamma n}} \frac {P({S_{n}}-E(S_{n})>x)}{n\overline{F}(x)} \leq c_{2} \bigl(L_{F}^{-1}+\varepsilon \bigr). \end{aligned}$$

By the arbitrariness of *ε*, it holds that

$$ \limsup_{n\to\infty}{\sup_{x\geq\gamma n}} \frac {P({S_{n}}-E(S_{n})>x)}{n\overline{F}(x)} \leq c_{2} L_{F}^{-1}. $$

This completes the proof of Theorem 2.2.

### Proof of Theorem 2.3

By Lemma 3.3, we know that Assumptions 1 and 4 are satisfied, and for any fixed $T>0$,

$$\limsup_{n\to\infty}{\sup_{x\geq T}}\frac{\sum_{i=1}^{n}\overline{F_{i}}(x)}{n{\overline{F}}(x)} \leq\limsup_{n\to\infty}{\sup_{x\geq0}} \frac{\sum_{i=1}^{n}\overline{F_{i}}(x)}{n{\overline{F}}(x)}=c_{6}. $$

Thus, by Theorem 2.2, for every fixed $\gamma>0$ and some fixed $T>0$,

$$\begin{aligned} \limsup_{n\to\infty}{\sup_{x\geq\gamma n}}\frac{P (S_{n} - E(S_{n})>x )}{n{\overline{F}}(x)} &\leq {L_{F}} ^{-1} \limsup_{n\to\infty}{\sup _{x\geq T}}\frac{\sum_{i=1}^{n}\overline{F_{i}}(x)}{n{\overline{F}}(x)} \\ &\leq c_{6}{L_{F}} ^{-1}. \end{aligned}$$

This completes the proof of Theorem 2.3.
